# Second-Degree Burns Following Intense Pulsed Light Therapy in a Patient With Fitzpatrick Skin Type IV: A Case Report

**DOI:** 10.7759/cureus.90119

**Published:** 2025-08-14

**Authors:** Jisu Kim, Matthew Cartier, Monica Morcos

**Affiliations:** 1 Internal Medicine, Hospital Universitario Dr. José Eleuterio González, Universidad Autónoma de Nuevo León, Monterrey, MEX; 2 Dermatology, Hospital Zambrano Hellion, Tecnológico de Monterrey, San Pedro Garza Garcia, MEX; 3 Mathematics, University of Pittsburgh, Pittsburgh, USA

**Keywords:** burns, fitzpatrick type iv, intense pulsed light, ipl complications, non-medical operator

## Abstract

Intense pulsed light (IPL) therapy is widely used in cosmetic dermatology for the treatment of unwanted hair, vascular lesions, pigmentary disorders, and acne-related sequelae. We present a case of a male in his late 20s with Fitzpatrick skin type IV who sustained second-degree burns following IPL therapy for post-acne erythema performed by a non-medical operator. The patient developed erythema, blisters, and desquamation. He was treated with oral medications (acyclovir and cephalexin), corticosteroid ointment, and medicated powder dressings. Subsequent treatment included light-emitting diode (LED) therapy, picosecond laser sessions, and topical depigmenting agents. Significant improvement was noted after four months with minimal residual hyperpigmentation. This case highlights the risks associated with IPL treatment administered by untrained personnel and underscores the importance of professional oversight and patient-specific parameter adjustments.

## Introduction

Intense pulsed light (IPL) is widely used in dermatology due to its versatility and efficacy. IPL devices emit polychromatic, high-intensity pulses of light that selectively target chromophores such as melanin and hemoglobin, making them effective for treating conditions like unwanted hair, vascular lesions, pigmentary disorders, and acne-related hyperpigmentation [1-3]. Although generally safe, IPL treatments can lead to adverse effects ranging from mild erythema to serious complications such as burns, blistering, and scarring [4-6]. IPL is most commonly used in individuals with Fitzpatrick skin types I to III and is generally discouraged in patients with darker skin or recent tanning, due to the increased risk of epidermal damage from competing melanin absorption [7]. With the growing popularity of IPL procedures, treatments are increasingly performed by non-physician operators, who account for a substantial proportion of cases involving adverse outcomes [8,9]. We present a case of a Fitzpatrick IV patient who developed second-degree burns following IPL treatment for acne sequelae at a non-medical spa, where the procedure was performed without appropriate medical supervision or individualized parameter adjustment.

## Case presentation

**Figure 1:**
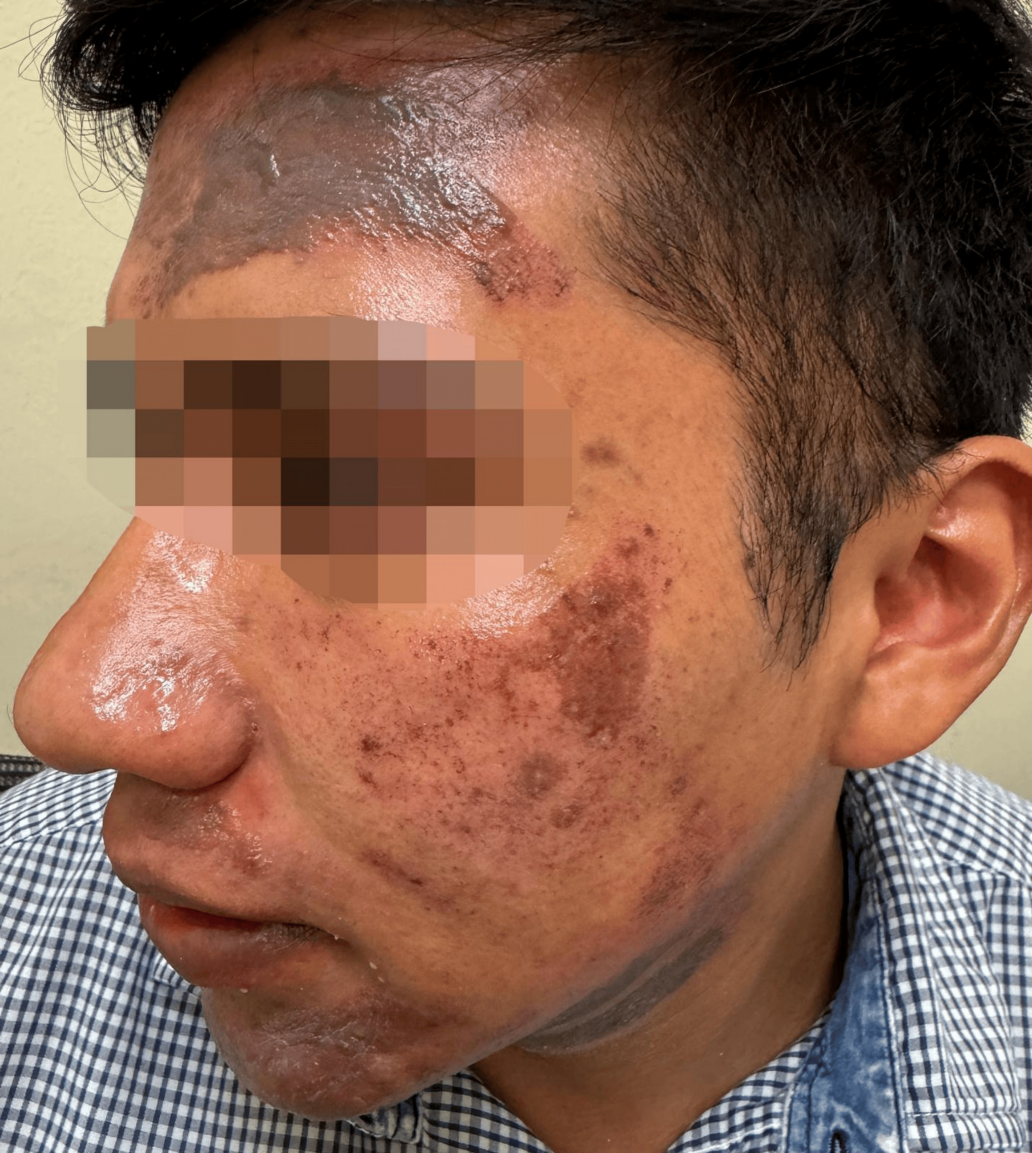
Erythematous, superficial partial-thickness burns were observed over the left forehead and zygomatic region, accompanied by post-inflammatory hyperpigmentation. The patient provided verbal and written informed consent for the publication of this case and any accompanying clinical images in an open-access format.

**Figure 2:**
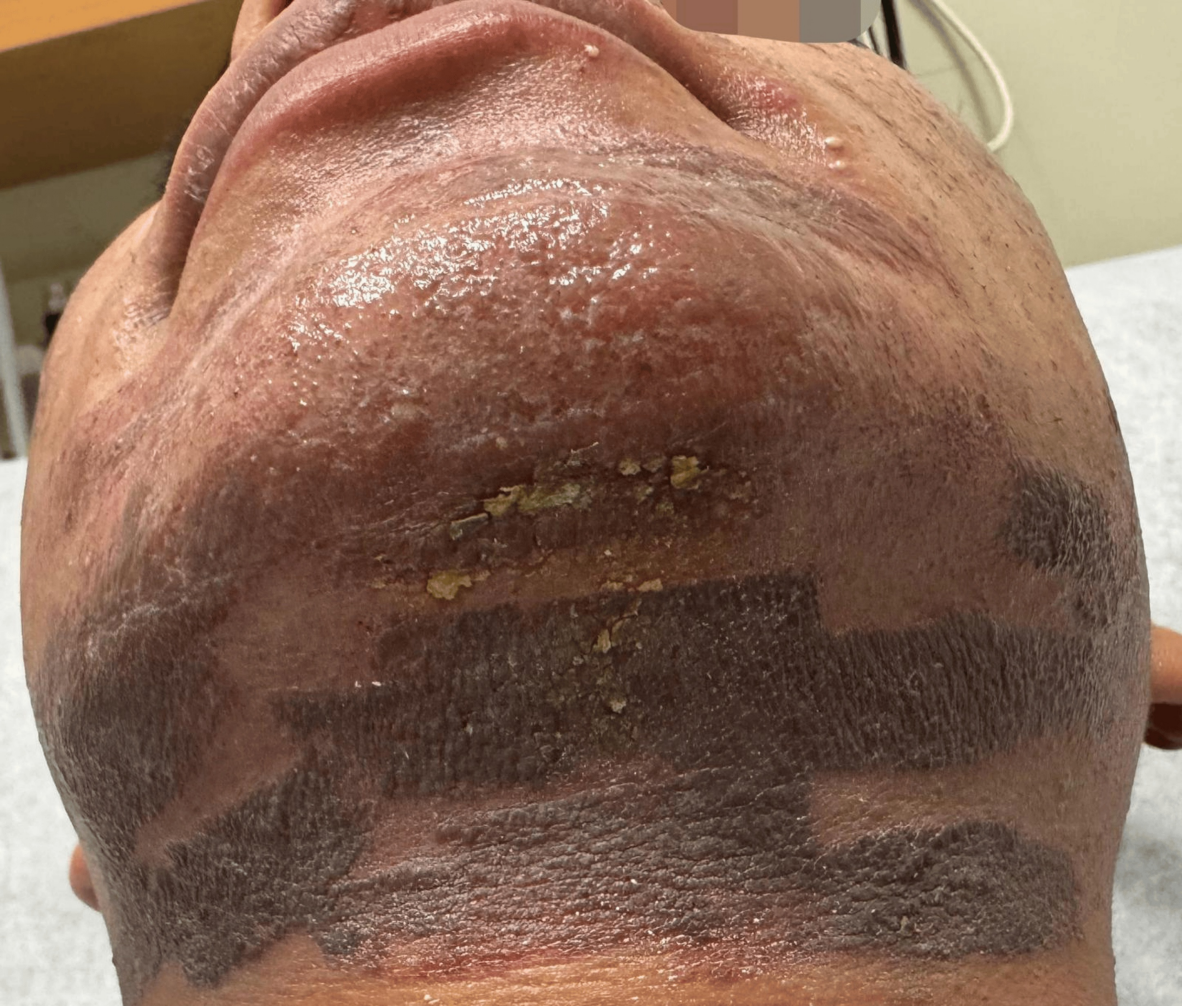
Desquamation and post-inflammatory hyperpigmentation were noted in the submental and submandibular regions, conforming to the shape of the IPL handpiece contact area. IPL: intense pulsed light

**Figure 3:**
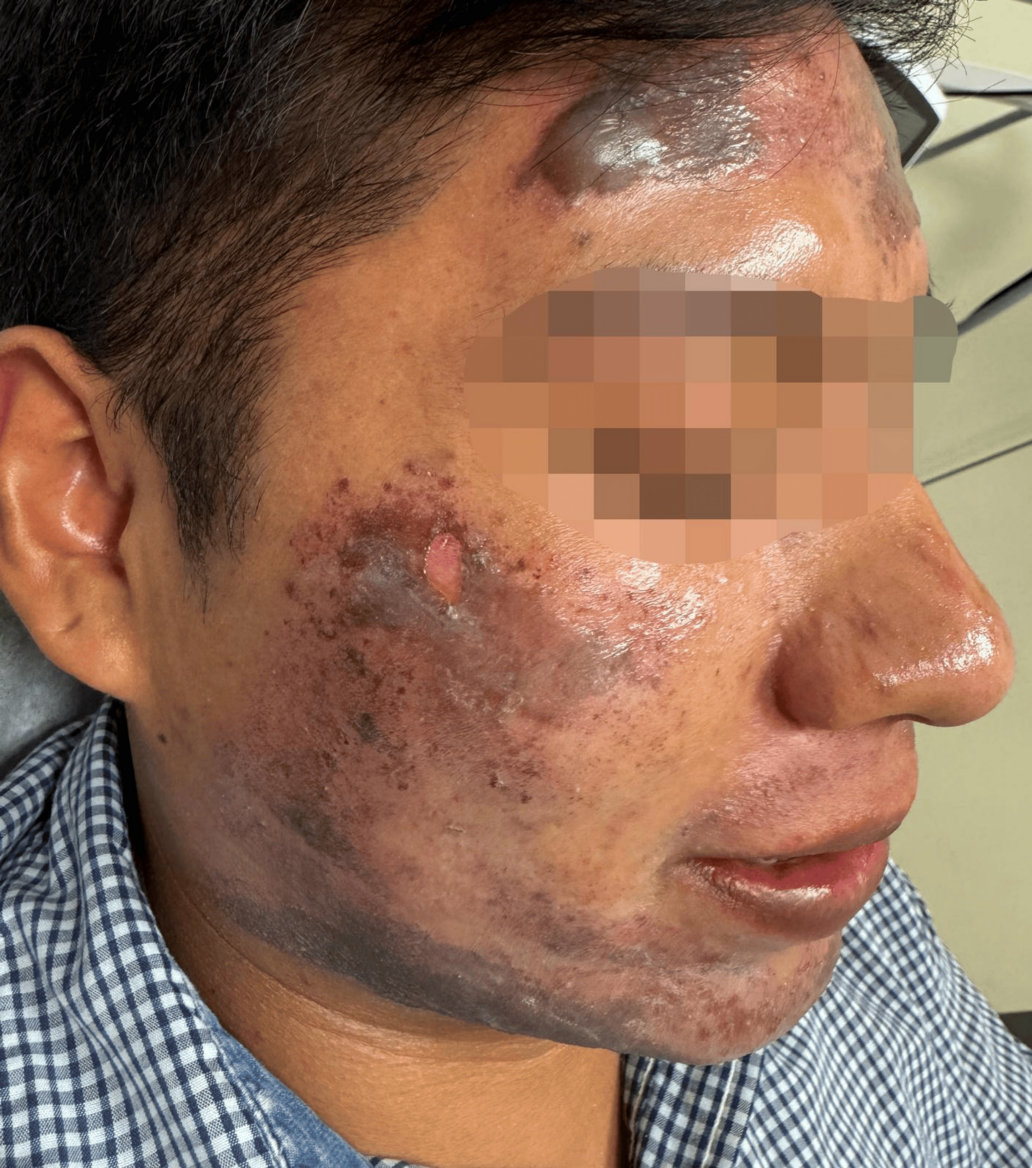
Superficial partial-thickness burns with tense bullae, erosions, and post-inflammatory hyperpigmentation over the right forehead and zygomatic region. The patient provided verbal and written informed consent for the publication of this case and any accompanying clinical images in an open-access format.

A male in his late 20s with Fitzpatrick skin type IV (light brown skin) presented to a private dermatology clinic with superficial and partial-thickness thermal burns on the face and neck after undergoing IPL treatment at a non-medical aesthetic spa. The procedure was conducted as part of a series for post-acne erythema and hyperpigmentation. During the session, the patient reported significant pain, but his complaints were dismissed, as it was his third treatment with the same settings. Post-procedure, he experienced persistent burning, discomfort, and blister formation, prompting him to seek urgent dermatological care (Figures 1-3).

Initial treatment included acyclovir, cephalexin, corticosteroid ointment, and a medicated powder dressing. The patient was advised to return after seven days. At follow-up, burn areas appeared pink and smooth, indicating epithelial regeneration. However, due to the risk of post-inflammatory hyperpigmentation, further treatment was initiated.

**Figure 4:**
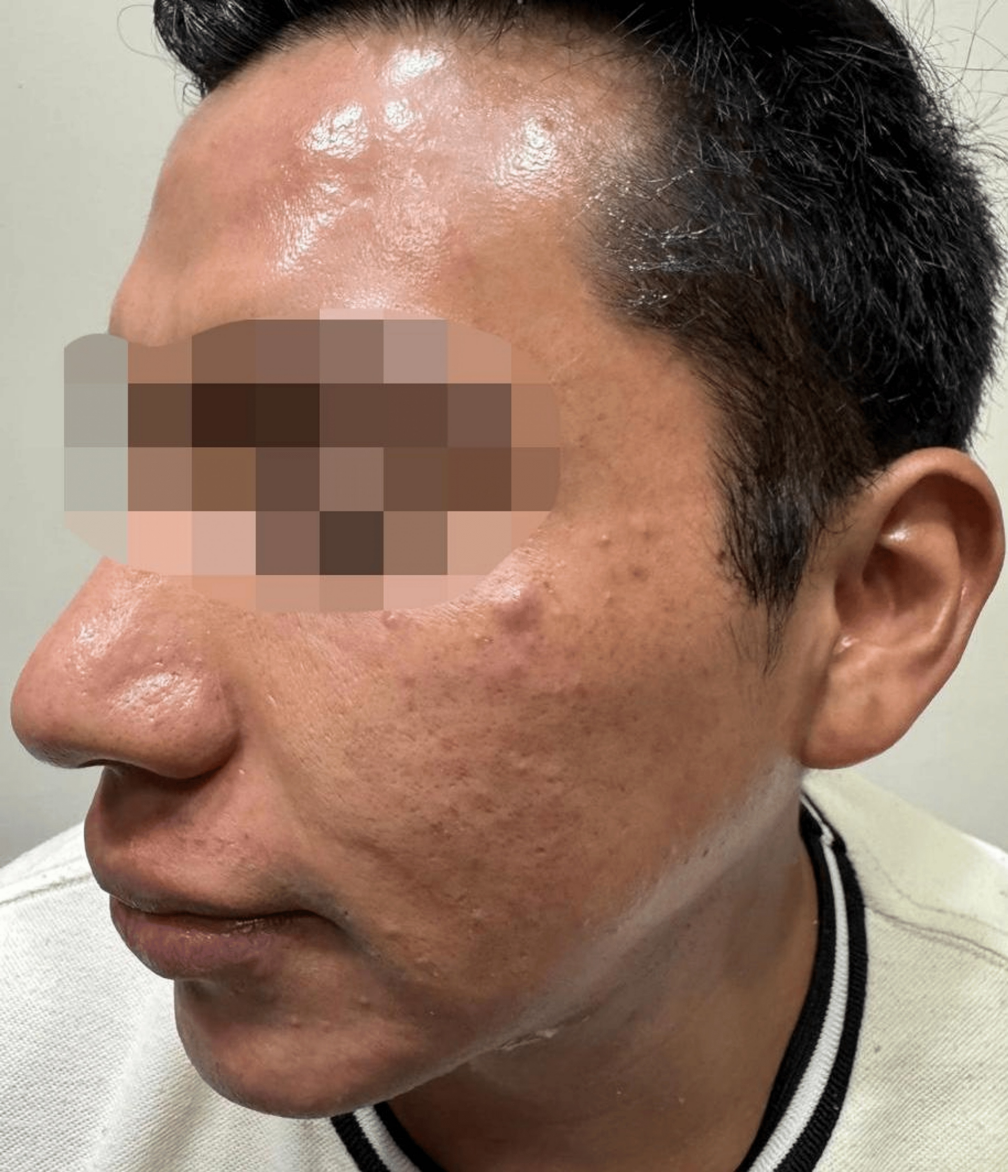
Left side at four-month follow-up showing smooth skin with resolution of desquamation and post-inflammatory hyperpigmentation. The patient provided verbal and written informed consent for the publication of this case and any accompanying clinical images in an open-access format.

**Figure 5:**
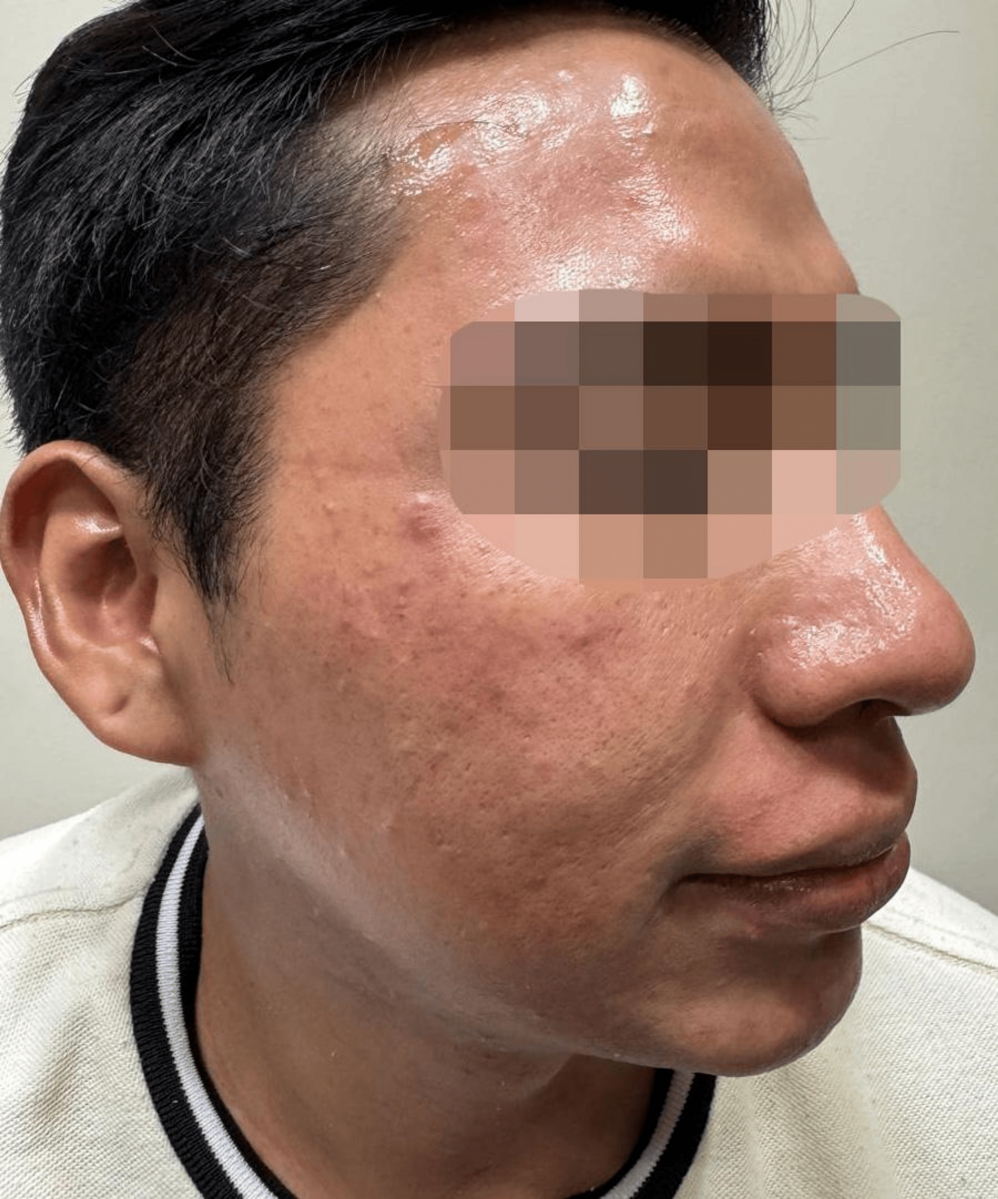
Right side at four-month follow-up showing resolution of bullae and vesicles with re-epithelialized skin and minimal residual post-inflammatory hyperpigmentation. The patient provided verbal and written informed consent for the publication of this case and any accompanying clinical images in an open-access format.

**Figure 6:**
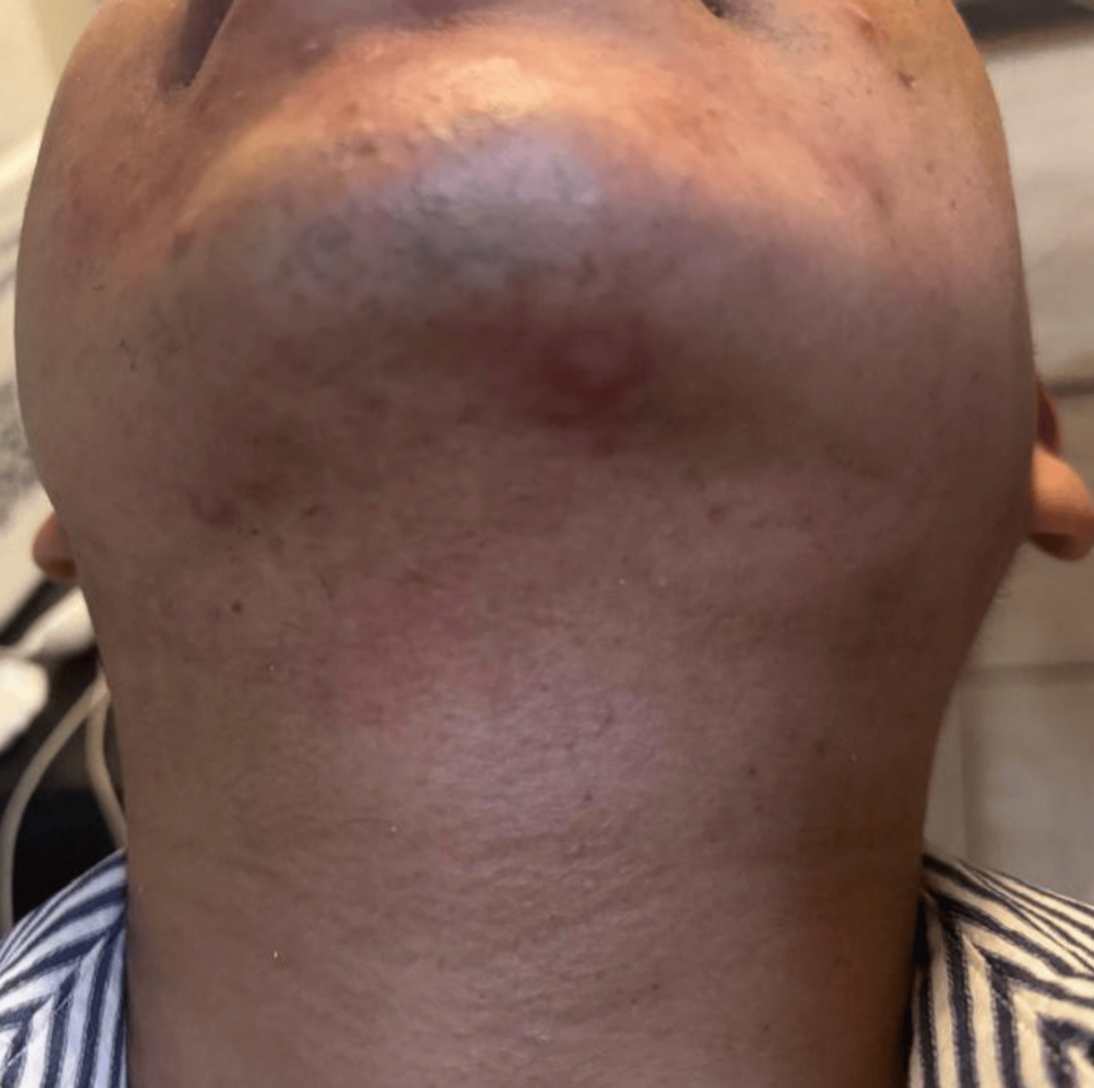
Submental region at four-month follow-up showing smooth, re-epithelialized skin with resolution of prior desquamation and post-inflammatory hyperpigmentation.

In subsequent visits, the patient received four sessions of LED phototherapy and three sessions of picosecond laser treatment, distributed over four months, along with topical depigmenting agents containing medical-grade azelaic acid and kojic acid, combined with pomegranate and garden cress extracts. The patient showed marked improvement with only minimal residual hyperpigmentation and no hypertrophic scarring (Figures 4-6).

## Discussion

Intense pulsed light (IPL) treatments can lead to burns, particularly in individuals with darker skin types, due to the broad absorption spectrum of melanin. In Fitzpatrick skin types IV to VI, the increased epidermal melanin competes with hemoglobin for light absorption, elevating the risk of unintended thermal injury [7]. Given these risks, IPL treatment is generally not recommended for patients with Fitzpatrick skin type higher than III [10]. When IPL is used, parameter selection must be tailored to each patient, accounting for variables such as skin tone, dermal thickness, and tanning history. Adequate cooling methods (such as cryogen spray, cooling gels, or chilled handpieces) are essential for minimizing epidermal damage [11].

In this report, the patient's Fitzpatrick IV skin type, combined with non-individualized IPL settings and the dismissal of pain during treatment, likely contributed to the development of second-degree burns. The procedure was performed in a non-medical setting without physician oversight.

In a randomized controlled trial involving 15 subjects with Fitzpatrick skin types II-IV, Thaysen-Petersen et al. evaluated IPL-induced side effects [12]. Adverse reactions were common and included erythema (87% of patients), hyperpigmentation (60%), purpura (27%), blisters (20%), hypopigmentation (20%), edema (13%), and crusting (13%). Notably, darker skin pigmentation and higher IPL fluence were both significantly correlated with more severe side effects (p≤0.002). These findings occurred even under standardized clinical protocols, highlighting the narrow therapeutic window in darker skin types.

In another study, Radmanesh et al. evaluated 2,541 women undergoing IPL hair removal in a large clinical series [6]. Although their study focused on hair removal and Fitzpatrick IV-V skin types represented only 28% of the cohort, these darker-skinned patients experienced higher rates of pigmentary changes and epidermal injury compared to lighter skin types, with a statistically significant correlation (p=0.001) between skin type and incidence of burning sequelae.

The risks associated with IPL treatments performed by non-medical practitioners, particularly in darker-skinned individuals, have been previously documented. Hammes et al. evaluated 43 patients who experienced complications after IPL and laser treatments administered by medical laypersons without physician oversight, finding pigmentary changes in 81.4% of cases, scarring in 25.6%, and textural changes in 14% [8]. Treatment errors primarily involved excessively high energy settings (62.8% of cases), inappropriate device selection (39.5%), and treating patients with darker skin or marked tanning (20.9%). These findings underscore the necessity for standardized protocols and direct physician involvement, particularly when treating patients with darker skin types.

While IPL presents increased risks in darker skin types, alternative laser modalities may offer safer options. Long-pulsed Nd:YAG lasers (1064 nm), which penetrate deeply and exhibit lower epidermal melanin absorption, are considered the safest and most effective for patients with Fitzpatrick skin types IV-VI, particularly in hair removal and vascular treatments [10,11]. Additionally, the use of higher wavelength cutoff filters, such as a 560 nm filter instead of the more melanin-absorptive 515 nm filter typically used in lighter-skinned patients, can reduce melanin interaction during IPL procedures, thereby minimizing the risk of adverse effects in patients with darker skin [7,10].

A notable strength of this report is the thorough clinical documentation, including follow-up treatment with LED therapy, picosecond laser, and topical depigmenting agents, which successfully minimized long-term sequelae. However, this remains a single case, limiting its generalizability. Additionally, no biopsy or histopathologic confirmation was performed, though the clinical features were consistent with partial-thickness thermal injury. An added limitation is the paucity of literature specifically addressing IPL-induced burns in darker-skinned patients treated for acne, which restricts direct comparisons and highlights the need for further research in this high-risk population.

With the growing availability of IPL in non-medical environments such as spas and beauty centers, this case underscores the need for stricter regulation, improved practitioner training, and public education regarding the risks of energy-based devices. While IPL is a valuable therapeutic modality, it requires informed judgment and technical skill, especially in darker skin types, where adverse outcomes are more likely if care is not properly individualized and supervised.

## Conclusions

Although IPL is often marketed as a cosmetic procedure, it is a medical device that requires clinical understanding of skin physiology and laser-tissue interactions. This case demonstrates the potential for serious complications, such as second-degree burns, when IPL is performed by non-medical personnel without adequate training. While not all aesthetic complications can be extrapolated globally, this case underscores the need for stronger regulatory frameworks and credentialing of operators in Mexico and other countries with rapidly growing aesthetic markets. Particular caution is warranted when treating patients with darker skin types, who face a significantly narrower margin of safety with IPL. Ensuring safe use of this technology depends not only on the device itself, but on informed clinical judgment and professional oversight.
